# Differences in predominant collagen fiber orientation between dorsal and plantar trabecular bone tracts of adult mule deer calcanei suggest strain‐mode‐specific adaptation

**DOI:** 10.1002/ar.70155

**Published:** 2026-02-11

**Authors:** John G. Skedros, John T. Cronin, Chad S. Mears, Brett W. Richards

**Affiliations:** ^1^ Department of Orthopaedic Surgery University of Utah Salt Lake City Utah USA; ^2^ Research Department Utah Orthopaedic Specialists Salt Lake City Utah USA; ^3^ Research Service Veterans Affairs Medical Center Salt Lake City Utah USA

**Keywords:** bone adaptation, cancellous bone, collagen fiber orientation, deer calcaneus, hemi‐osteons, trabecular bone

## Abstract

Studies examining biomechanics of trabecular (cancellous) bone often focus on its architectural characteristics, including bone volume fraction, degree of preferred trabecular alignment, and morphology of individual trabeculae. Studies examining relationships of these characteristics with load history in specific regions of limb bones usually aim to identify species affiliations or how these characteristics affect mechanical properties. This focus on structural characteristics seems warranted because >70% of variance in strength tests of bulk trabecular bone specimens is explained by structural characteristics. However, biomechanically significant differences in trabecular bone material characteristics may be consequently overlooked. We examined this possibility by quantifying predominant collagen fiber orientation (CFO) in thin transverse sections from adult deer calcanei at mid‐shaft. This bone has simple load history, producing net compression in its dorsal aspect and net tension in its plantar aspect. Predominant CFO was quantified in circularly polarized light images as weighted mean gray levels (WMGLs) of the (1) dorsal “compression cortex” and plantar “tension cortex,” and (2) dorsal and plantar trabecular tracts (presumably also net compression‐ vs. tension‐loaded). Brighter gray levels are expressed as higher WMGLs and reflect compression‐adapted CFO; darker gray levels reflect tension‐adapted CFO. Results showed more oblique‐to‐transverse CFO in the dorsal trabecular bone and longitudinal in the plantar trabecular bone (dorsal 126.6 ± 18.6; plantar 98.3 ± 16.2; *p* < 0.01). Although this difference resembles strain‐mode‐specific (compression vs. tension) adaptation in the dorsal/plantar cortices, adequate exploration of this hypothesis in trabecular bone will require mechanical testing coupled with micro‐ and nano‐scales analyses in this model and in other bones in other species.

## Graphical abstract

Polarized microscopic images of the outer bone cortex (a and b) and deeper trabecular bone (c and d) of the deer calcaneus in thin cross‐sections. The brighter gray levels reflect more oblique‐to‐transverse collagen fibers in the compression/dorsal bone (a, c) and the darker gray levels reflect more longitudinal collagen in the tension/plantar bone (b, d).The hypothesis that these gray level differences represent adaptation for compression vs. tension is an intriguing possibility worthy of additional investigation.

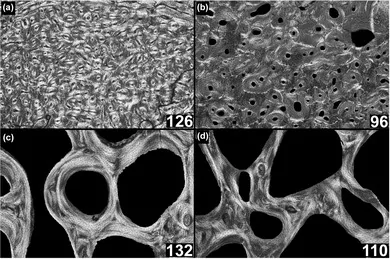

## 1 INTRODUCTION

The artiodactyl (wild deer and domesticated sheep) calcaneus model is useful for bone adaptation studies because of its relatively simple load history, which is characterized by sagittal‐plane bending resulting in regions that independently experience net compression versus net tension (Skedros et al., 2004; Skedros et al., 2019; Skedros, Dayton, Bloebaum, et al., 2024) (Figure 1). This load history has been documented by in vivo and rigorous ex vivo analyses that have employed mechanical strain gauges attached directly on the bone, allowing for direct measurements of deformation (e.g., compression, tension, and shear strains) (Lanyon, 1973, 1974; Skedros et al., 2019). These calcanei have been used as comparative “control bones” for basic and clinical studies involving the load history of the human femoral neck (Skedros, 2025a), which is classically conceived as receiving habitual unidirectional bending—much like the artiodactyl calcaneus (Bell et al., 1999; Sinclair et al., 2013; Skedros & Baucom, 2007). In deer and sheep calcanei and other limb bones (e.g., radii of sheep and horses), regional variations in predominant collagen fiber orientation (CFO) in *cortical* (compact) bone have been shown to be strong correlates of a predictable unidirectional bending environment where there is net compression on one side of the bone and net tension on the other during weight‐bearing locomotion (Mason et al., 1995; Skedros et al., 2009). This strong relationship between load history and predominant CFO is especially important for interpreting the load history of a bone region where, like the human femoral neck, the use of strain/deformation gauges is not possible due to ethical or technical concerns. Furthermore, the structural and material characteristics of deer and sheep calcanei have been rigorously described compared to the human femoral neck (Skedros, 2025a; Skedros et al., 2023). Patterns of predominant CFO in trabecular bone, however, have not been reported for the deer and sheep calcanei.

**FIGURE 1:**
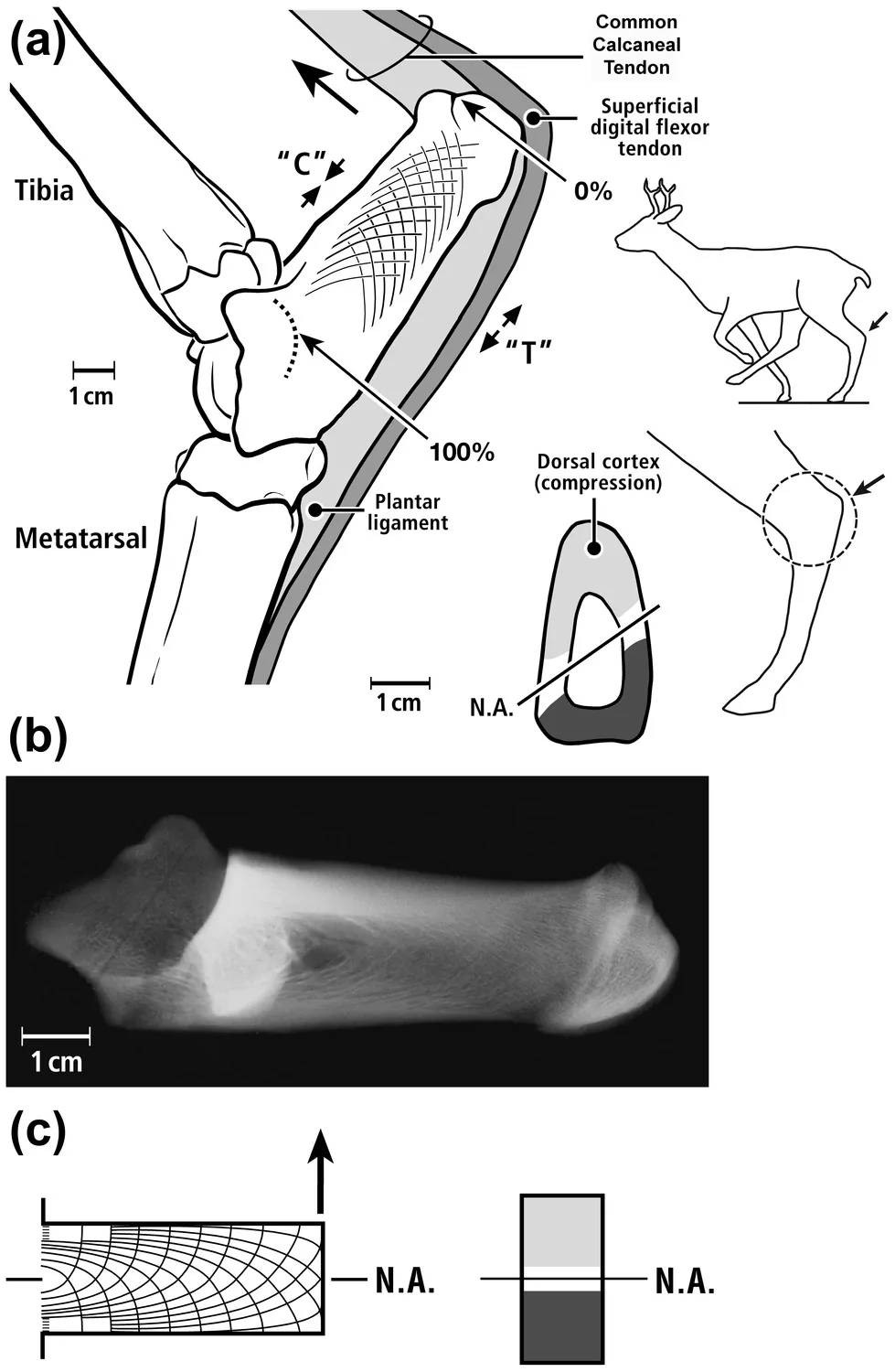
Lateral view of left adult mule deer and deer calcaneus in enlarged view of the ankle (hock) region (a), lateral radiograph of an adult mule deer calcaneus (b), and an idealized cantilevered beam (c). (a) At the right, the small arrows point to the location of the distal portion of the calcaneus in the drawing of the entire animal and in an outline drawing showing the hind limb and ankle (hock) region (circled). The large arrow in the enlarged drawing at the left shows the direction of force applied to the calcaneus via the common calcaneal (“Achilles”) tendon during a portion of the stance (weight‐bearing) phase; this loads the dorsal cortex in dominant compression (“C”) and plantar cortex in dominant tension (“T”). The arched trabecular patterns are shown in a stylized/simplified fashion that approximate the theoretical stress trajectories of a trajectorial structure (Skedros & Baucom, 2007). At the right is a transverse section from the proximal third of the calcaneal shaft, and the obliquity of the neutral axis (N.A.) is shown (Su et al., 1999). (b) Lateral radiograph of an adult mule deer calcaneus showing arched trabecular patterns. (c) Idealized cantilevered beam, end‐loaded in bending, with orthogonally‐intersecting theoretical stresses trajectories depicted by curved lines. Notably, the arched patterns in the idealized cantilevered beam and lateral radiograph resemble one another. On the right is a transverse section of the beam showing its horizontal neutral axis (N.A.), which is what is expected in an idealized end‐loaded cantilevered beam (Reproduced from Skedros et al. (2023, 2012, 2019) with permission of John G. Skedros).

Similarly, there is a paucity of literature that has quantified predominant CFO patterns in the trabecular bone of the human femoral neck. The only study that we are aware of in the context of the femoral neck is that of von Kroge et al. (2022) who reported an unexpected lack of CFO differences between the presumed “tension” and “compression” trabecular tracts of the human femoral neck. But, as argued below, the complex/multi‐directional loading of the femoral neck likely blunts simple strain‐mode relationships. In this perspective, our study focused on quantifying relative regional differences in predominant CFO in the putative “tension trabecular tract” and “compression trabecular tract” of the comparatively simply loaded deer calcaneus model. We predicted: (1) more oblique‐to‐transverse “compression‐adapted” CFO in the dorsal than plantar trabecular tracts, and (2) greater CFO‐heterogeneity in the dorsal than plantar tracts. In this study, weighted mean gray levels (WMGLs) from circularly polarized light (CPL) images are used as a proxy for predominant CFO. CFO‐heterogeneity is used to indicate the spread of the gray‐level distribution reflecting CFO dispersion.

A very large majority of the studies on the adaptability of trabecular bone have focused on its structural (architectural) variations (Kivell, 2016; Reznikov et al., 2016; Saers et al., 2022a, 2022b; Salmon et al., 2015; Sinclair et al., 2013). The reasons for this focus are clear—a large percentage of variance in conventional mechanical tests of trabecular bone bulk specimens can be explained by variations in a few of these *architectural* characteristics (e.g., >70% of the variance in strength tests is often explained by bone volume fraction, the degree of preferred trabecular alignment, and the morphology of individual trabeculae) (Diederichs et al., 2009; Goldstein et al., 1993; Goulet et al., 1994; Keaveny et al., 2001; Liu et al., 2010; Mittra et al., 2005; Perilli et al., 2008; Sinclair et al., 2013; Ulrich et al., 1999). However, there is evidence that trabecular bone can also exhibit phenotypic plasticity in its material organization, which may have important influences on less studied, but clinically/biomechanically relevant, mechanical properties (e.g., energy absorption and fatigue resistance). These material variations include: (1) bone tissue mineral density, (2) bone tissue mineral density distribution (a measure of mineralization heterogeneity), (3) hemi‐osteonal “packet” prevalence (defined below), (4) lamellation differences in hemi‐osteonal packets, (5) nanoscale mineral grain patterns, (6) collagen cross‐links, and (7) variations in collagen/lamellar organization (including fibrillar/fiber alignment) (Busse et al., 2009; Carretta et al., 2015; Ciarelli et al., 2009; Hernandez et al., 2005; Lamarche et al., 2025; Lozupone, 1985; Milovanovic et al., 2012; Reznikov et al., 2015; Roschger et al., 2008; Skedros et al., 2012; Torres et al., 2016; Turunen et al., 2020; von Kroge et al., 2022). Variations in the material characteristics of cortical and trabecular bone are often mediated by the remodeling process, which refers to the formation of secondary osteons (Haversian systems). It is the process whereby a small cavity of bone tissue (~150–200 μm in diameter) is first resorbed by osteoclasts, and then is followed by filling of the cavity through coupled osteoblastic bone formation (Martin et al., 2015). The remnants of remodeling cycles are quasi cylindrical osteons in cortical (compact) bone; in trabecular bone they are “hemi‐osteons,” which are produced by semi‐concentric deposition of lamellar bone (Martin et al., 2015; Parfitt, 1994).

The basic structural units typically formed by hemi‐osteons within trabecular bone are referred to as “packets” (Ciarelli et al., 2009; Lamarche et al., 2025; Parfitt, 1979; Skedros et al., 2012). The possibility that trabecular bone tissue can exhibit mechanically adaptive variations in the collagen/lamellar organization of these “packets” has received little attention. In contrast, it is well known that cortical bone has the capacity to adapt in terms of the collagen/lamellar organization of secondary osteons that are analogous to the hemi‐osteonal packets of trabecular bone (Martin et al., 1996; Skedros, 2025b). In cortical bone these are called secondary osteon collagen/lamellar “morphotypes,” which are important toughening mechanisms, especially for the nonuniform strain distributions (i.e., tension vs. compression strains) that typically occur in the diaphyses of limb bones (Skedros, Dayton, Cronin et al., 2024; Skedros et al., 2013; Skedros, Kiser, et al., 2011; Skedros et al., 2009). Prior studies of the structural and material organization of trabecular bone in the deer calcaneus model have shown that there are significant differences in hemi‐osteonal packet prevalence (defined below), tissue mineralization, and tissue mineralization heterogeneity between the dorsal “compression region” and plantar “tension region” trabecular tracts (Figure 2) (Sinclair et al., 2013; Skedros et al., 2012). In those studies it is suggested that biomechanically significant differences between the dorsal (“compression”) and plantar (“tension”) regions reflect the mechanistic hypothesis that trabecular bone can adapt in accordance with the local predominant strain mode and magnitude (Sinclair et al., 2013; Skedros et al., 2012). This hypothesis is based on studies of bovine and human trabecular bone showing that:

1. energy absorption to yield, and ultimate and yield strengths are nearly 30% lower in tension versus compression loading (Bayraktar et al., 2004; Keaveny, Guo, et al., 1994; Keaveny, Wachtel, et al., 1994),
2. trabecular bone is weaker in tension than compression (Keaveny et al., 2001; Mitton et al., 1997; Sanyal et al., 2012),
3. yield strains in compression (0.2% offset criterion) are higher than those in tension (Keaveny, Wachtel, et al., 1994; Kopperdahl & Keaveny, 1998; Rincon‐Kohli & Zysset, 2009; Wolfram et al., 2011),
4. microdamage initiation and propagation, and morphology, occur differently and asymmetrically in tension and compression (Boyce et al., 1998; Nyman et al., 2009; Reilly & Currey, 1999; Wu & Niebur, 2012), where yielding at local principal stresses occurs in the range of 88–121 MPa for compression and 35–43 MPa for tension (Jungmann et al., 2011; Nagaraja et al., 2005),
5. trabecular architecture is highly sensitive to changes in predominant load direction and magnitude (Barak, 2024; Barak et al., 2011; Pontzer et al., 2006; van der Meulen et al., 2006; van der Meulen et al., 2009), and
6. when single trabeculae are subjected to loading, the mechanical properties are mostly affected by their material‐level properties (Carretta et al., 2015; Carretta, Luisier, et al., 2013; Carretta, Stüssi, et al., 2013; Frank et al., 2018; Hernandez et al., 2005; Jungmann et al., 2011).

**FIGURE 2:**
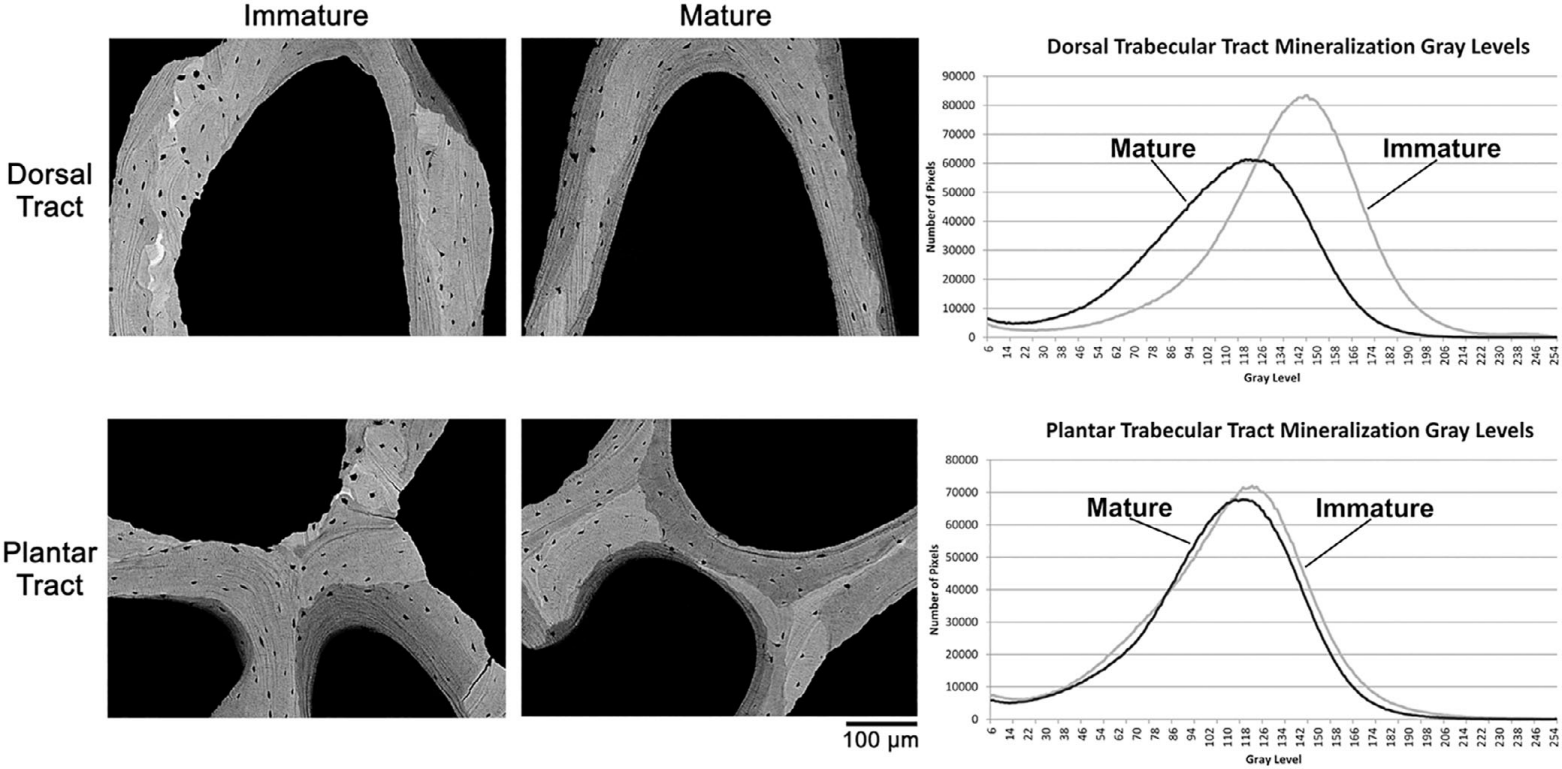
Backscattered electron images showing mineralization heterogeneity in trabecular bone of an adult deer calcaneus. These backscattered electron (BSE) images are from our prior study (Skedros et al., 2012) and were obtained from transverse sections of the mid‐shaft region of calcanei from immature (1–2 year old) and adult (3–4 year old) animals. The contralateral bones from the mature animals were used in the present study. In contrast to gray levels obtained from CPL images that represent variations in CFO, gray levels in BSE images represent differences in mineral content (Bloebaum et al., 1997). Images from immature and mature bones shown here illustrate growth‐related changes between the dorsal and plantar trabecular regions in mineralization heterogeneity (significant only in the immature bones) and average mineralization (significant in both immature and mature bones). These differences are shown graphically in the gray‐level profiles at the right of the images. Gray‐level profiles that are shifted more to the right (i.e., brighter gray levels) represent relatively higher mineralization (Bloebaum et al., 1997) (Reproduced from Skedros et al. (2012) with permission of John G. Skedros).

Similar to what has been reported in cortical bone, we hypothesize that trabecular bone has the capacity for comparable adaptations between regions that are independently subjected to ambient tension or compression strains. We suspect that this will be revealed in a bone, such as the deer calcaneus, that is habitually loaded in unidirectional bending where net compression and net tension are believed to occur in different regions of the trabecular bone during peak loading (Skedros et al., 2019). We predict that the dorsal trabecular tract will have more oblique‐to‐transverse CFO (compression adapted), and the plantar tract will have comparatively more longitudinal CFO (tension adapted), similar to the CFO differences reported previously between the opposing dorsal and plantar cortices of this model.

## 2 MATERIALS AND METHODS

This study was performed with approval of the Institutional Animal Care and Utilization Committee at the University of Utah (for the Orthopaedic Bioengineering Research Laboratory, protocol no. 09‐06005). The calcanei from each of 10 adult male mule deer (*Odocoileus hemionus hemionus*) were obtained from the contralateral limbs of the wild animals used in our prior studies of trabecular bone of this model (Sinclair et al., 2013; Skedros et al., 2012). From each bone, one 5 mm thick segment was cut transversely from the 50% to 55% location of the calcaneal shaft (tuber) (100% is nearest to the talus‐calcaneal joint) (Figure 3). The present analysis focused exclusively on the 50% to 55% location (and not more distally) because the loading environment is relatively simpler dorsal‐plantar bending in the central portion of the shaft (Skedros et al., 1994; Skedros et al., 2019). The segments were not stained or decalcified and were embedded in polymethyl methacrylate and prepared for imaging in accordance with published methods (Skedros et al., 2009). One surface of each embedded segment was ultramilled to a glass‐like finish and then was attached to a glass slide with cyanoacrylate glue; the opposite surface was then ultramilled to achieve a final 50 μm thickness. In order to reduce artifact that might result from sampling adjacent hemi‐osteons (as described further below), the thickness of 50 μm was one‐half of what we have used in prior studies.

**FIGURE 3:**
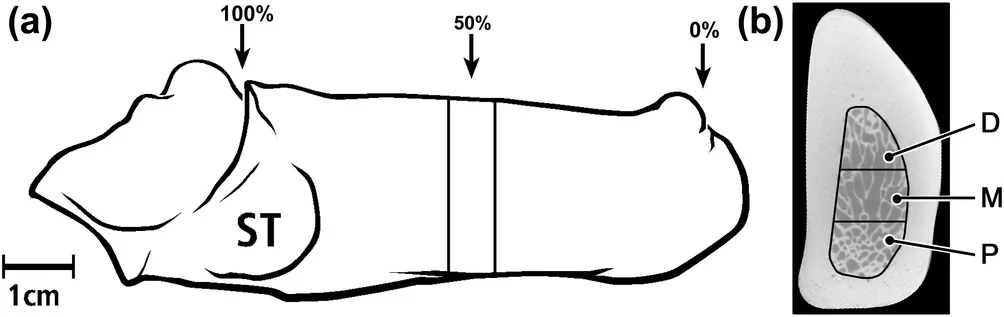
(a) Lateral view of a deer calcaneus showing the location sampled in this study (vertical lines). ST, sustentaculum talus. (b) Cross‐section of a deer calcaneus taken at the 50% location showing the distribution of cortical and trabecular bone. The trabecular bone can be divided into three regions: Dorsal (D), middle (M), and plantar (P). In this study, the CFO/WMGL analysis was conducted only in the dorsal and plantar trabecular bone regions (Reproduced from Sinclair et al. (2013) with permission of John G. Skedros).

The trabecular bone region of each section was subdivided into three smaller regions, where the middle region (not sampled) is where the dorsal and plantar tracts intersect extensively (Figure 3) (Sinclair et al., 2013). This resulted in similar‐sized areas of the cross‐section for analysis of trabecular bone in the plantar and dorsal regions. In each of these regions, 2–3 non‐overlapping fields were acquired, covering approximately 60%–70% of the mineralized tissue area. Fields were selected using a consistent judgment‐based approach that prioritized representative tissue morphology while avoiding sectioning artifacts. Digitized images were collected using a 4×/0.10 NA objective. The area of each image was 2.195 mm by 1.455 mm (4928 pixels by 3265 pixels; ~2.25 μm/pixel). The condenser was centered and opened until the field of view was evenly illuminated. As described previously (Skedros et al., 2009), polarized contrast was generated using appropriately crossed Polaroid filters that remained constant for all slides, with no retarder used. Lamp intensity was set using the microscope's “Photo” mode, which applies a fixed maximum illumination level. A Canon 80D camera body (lens removed) was mounted directly to the microscope, and all camera settings were held constant across specimens. White balance was set to 4700 K, and shutter speed was set to 1/800, while exposure and gain were unchanged. All imaging for the entire study was performed in a single session to eliminate day‐to‐day variation in illumination, alignment, or camera behavior.

Regional (dorsal vs. plantar) differences in CFO were inferred from corresponding differences in the intensity of transmitted light (based on the relative intensity of birefringence) (Skedros et al., 2009; Skedros et al., 2013). Digitized CPL images with 256 distinct gray levels (8‐bit depth) were used to determine a WMGL from each image as described in prior studies (Bloebaum et al., 1997; Skedros et al., 1996; Vajda et al., 1998). For the present study, as done in our prior CFO studies using CPL in thin sections of cortical bone (Skedros et al., 2009; Skedros et al., 2013), this method eliminates the darkest gray‐level values (i.e., values ≤20), which include tissue porosity and voids such as vascular canals, osteocyte lacunae, and non‐bone spaces (e.g., gross porosity of trabecular bone). These porous spaces are filled with the embedding material and appear dark/non‐birefringent in CPL images. For each microscopic image, a WMGL was calculated from the numerical gray‐level values of the image pixels (representing mineralized bone) using this equation (Bloebaum et al., 1997):

$$\text{WMGL}=\sum_{I=21}^{255}\frac{A_{i}{\mathrm{GL}}_{i}}{A_{t}}$$

where, *A*_*i*_ = area of the *i*th gray level; *GL*_*i*_ = *i*th gray level; and *A*_*t*_ = total area of trabecular bone in each CPL image.

As in our prior studies of cortical bone adaptation, brighter gray levels (i.e., greater birefringence) are expressed as higher WMGLs and suggest “compression‐adapted” CFO, whereas darker gray levels are expressed as lower WMGLs and suggest “tension‐adapted” CFO (Skedros et al., 2009; Skedros et al., 2013; Skedros, Kiser, et al., 2011). The use of gray scale in CPL images to evaluate CFO in thin sections of bone has been validated by independent laboratories (Boyde & Riggs, 1990; Bromage et al., 2003). Compression‐adapted CFO refers to collagen fibers with relatively more oblique‐to‐transverse orientation with respect to the long axis of the calcaneal shaft, and tension‐adapted CFO represents collagen fibers that are relatively more longitudinal with respect to the long axis of the calcaneal shaft (Boyde & Riggs, 1990; Skedros et al., 2009). The heterogeneity of CFO (CFO‐het) in each CPL image of trabecular and cortical bone was expressed as the width at one‐half of the maximum of the gray‐level histogram profile, which is similar to methods used to measure bone mineral density distributions in backscattered electron images (Roschger et al., 2008; Skedros et al., 2012; Skedros et al., 2014) (Figure 2).

Within individual trabeculae there are typically many hemi‐osteon “packets,” and they can be distinguished from each other by differences in gray levels in the CPL images. Hemi‐osteonal “packet prevalence” is expressed as the number of packets divided by the absolute area of the mineralized trabecular bone in each image. In the present study (as described below), these data are used in correlation analyses with CFO/WMGL and CFO‐het data and they are also summarized in box plots. Data from our prior study (Skedros et al., 2012) are used in correlation analyses with CFO/WMGL and CFO‐het data, and they are also summarized in box plots.

In the present study, a confocal microscope (PCM‐2000, Nikon, Melville, New York, USA) was used to examine seven indiscriminately selected hemi‐osteons in the dorsal and plantar trabecular regions from several of the 50 μm thick sections to verify that there was minimal overlap between adjacent packets. This was a qualitative assessment and was done by one investigator. This was done to ensure that the overlap of adjacent hemi‐osteonal packets, which might have significantly different collagen/lamellar organizations, was not averaged in the single WMGL value that was obtained for each image (as this could confound the data). Such overlap might occur because, unlike backscattered electron imaging that samples only a few microns of the bone surface (Skedros et al., 2005), CFO/CPL analyses are based on transmitted light that passes through the entire prepared section. Hence, these CFO measurements are based on an averaged gray‐level value measurement that could be artifactually obscured if adjacent packets with very different collagen/lamellar patterns are sampled simultaneously. This potential problem of “projection artifact” has been recognized in CFO studies of osteons in cortical bone when the section is not sufficiently thin (Bromage et al., 2003). This confocal assessment revealed that ~4.5% (14/303) of the examined hemi‐osteons overlapped with small portions of other hemi‐osteons. This supports the conclusion that the regional differences in CFO‐het and predominant CFO between the trabecular tracts reported in this study occur at the individual hemi‐osteonal level of tissue organization.

A power analysis was performed to estimate the sample sizes required to detect differences in predominant CFO that reflect statistically significant and mechanically important differences between cortical bone regions subject to habitual tension vs. compression (such data are not available for trabecular bone). We used predominant CFO data from CPL images from thin micro‐milled sections of plantar and dorsal cortices of adult mule deer (dorsal CFO/WMGL 135 ± 6, plantar CFO/WMGL 101 ± 16) (Skedros et al., 2009). It was determined that a sample size of at least eight bones in each group would achieve 95% power (with *α* = 0.05) to detect a CFO/WMGL difference of that magnitude. Although we assumed that a similar sample size would be sufficient for detecting statistically significant and mechanically important differences in regional CFO‐het in the deer calcaneus, this was uncertain due to a lack of comparable data for cortical or trabecular bone.

The CFO/WMGL and CFO‐het data obtained in this study are expressed as means, medians, and standard deviations. For all statistical procedures, the NCSS statistics package was used (NCSS 2020, NCSS LLC, Kaysville, Utah, USA; NCSS.com). Paired *t*‐tests were conducted to determine if there are significant differences in predominant CFO/WMGL and CFO‐het data between the dorsal and plantar trabecular tracts and cortices. Statistical significance was set at *p* values ≤0.05. Pearson correlation coefficients were calculated for paired comparisons of data from the dorsal and plantar trabecular bone regions to determine if there are potentially biomechanically important relationships among the various material characteristics reported herein and previously (Skedros et al., 2012). In that prior study, average mineralization was quantified as WMGLs in backscattered electron images of the cortical bone in each region using the same calculation used to determine CFO/WMGL described above. These correlation analyses were conducted on five material characteristics, including CFO/WMGL and CFO‐het data obtained from the present study and the mean mineral content (average mineralization), mineral content heterogeneity, and hemi‐osteonal packet prevalence data also from that prior study. These three non‐CFO characteristics had been obtained from backscattered electron images of the same embedded tissue segments used in the present study. Correlations were not conducted with the structural data reported previously (Sinclair et al., 2013; Skedros et al., 2012) because those data were obtained from different animals.

## 3 RESULTS

Results of the analysis of cortical bone show the expected differences in CFO (expressed as WMGL): more oblique‐to‐transverse “compression‐adapted” CFO in the dorsal cortex vs. more longitudinal “tension‐adapted” CFO in the plantar cortex (dorsal CFO/WMGL 119.8 ± 6.3; plantar CFO/WMGL 91.8 ± 5.3; *p* < 0.01). Supporting the main hypothesis of this study regarding variations in trabecular bone, CFO is also more oblique‐to‐transverse in the dorsal trabecular bone and more longitudinal in the plantar trabecular bone (dorsal CFO/WMGL 126.6 ± 18.6; plantar CFO/WMGL 98.3 ± 16.2; *p* < 0.01) (Figure 4). Thus, the dorsal/plantar CFO/WMGL difference is 31% for the cortical bone and 29% for the trabecular bone; in both cases there is more oblique‐to‐transverse CFO in the dorsal regions (Figure 5). The mean and median values shown in Figure 5a for dorsal/plantar CFO ratios for trabecular bone are 1.29 and 1.30, respectively. CFO‐het is greatest in the dorsal (“compression region”) trabecular bone when compared to the plantar trabecular bone (*p* < 0.05) (Figure 5). The mean and median values shown in Figure 5a for dorsal/plantar CFO‐het ratios for trabecular bone are 1.23 and 1.21, respectively.

**FIGURE 4:**
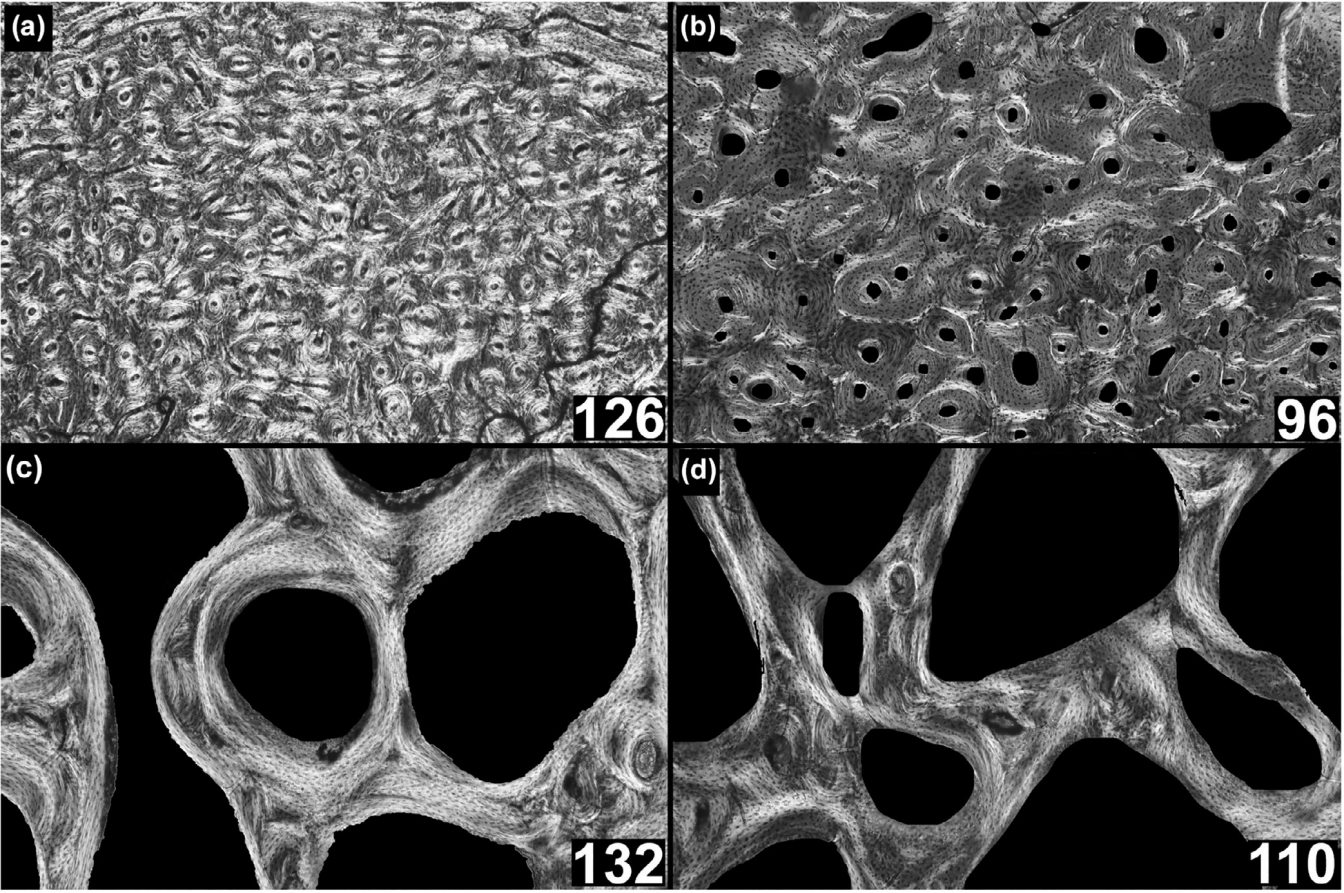
CPL images obtained in the present study from the same calcaneus in these regions: Dorsal cortical bone (a) and dorsal trabecular bone (c), and plantar cortical bone (b) and plantar trabecular bone (d). These images are representative of the bones used in this study. At the bottom right in each image is the weighted mean gray level (WMGL) that was obtained for the bone in each image. (a) and (c) demonstrate brighter gray levels that represent more oblique‐to‐transverse in the dorsal region of the calcaneus; (b) and (d) show darker gray levels that represent more longitudinal CFO in the plantar region. The width of each image is 2.195 mm. In post‐image processing, the porous spaces in these digitized images have been made black (i.e., gray level “0”) in order to enhance the visualization of the bone. Discussion regarding the biomechanics and physiological implications of the dorsal versus plantar differences in osteon size, population density, and osteonal remodeling rates can be found in our prior studies (Skedros et al., 2001; Skedros et al., 2004; Skedros et al., 2013).

**FIGURE 5:**
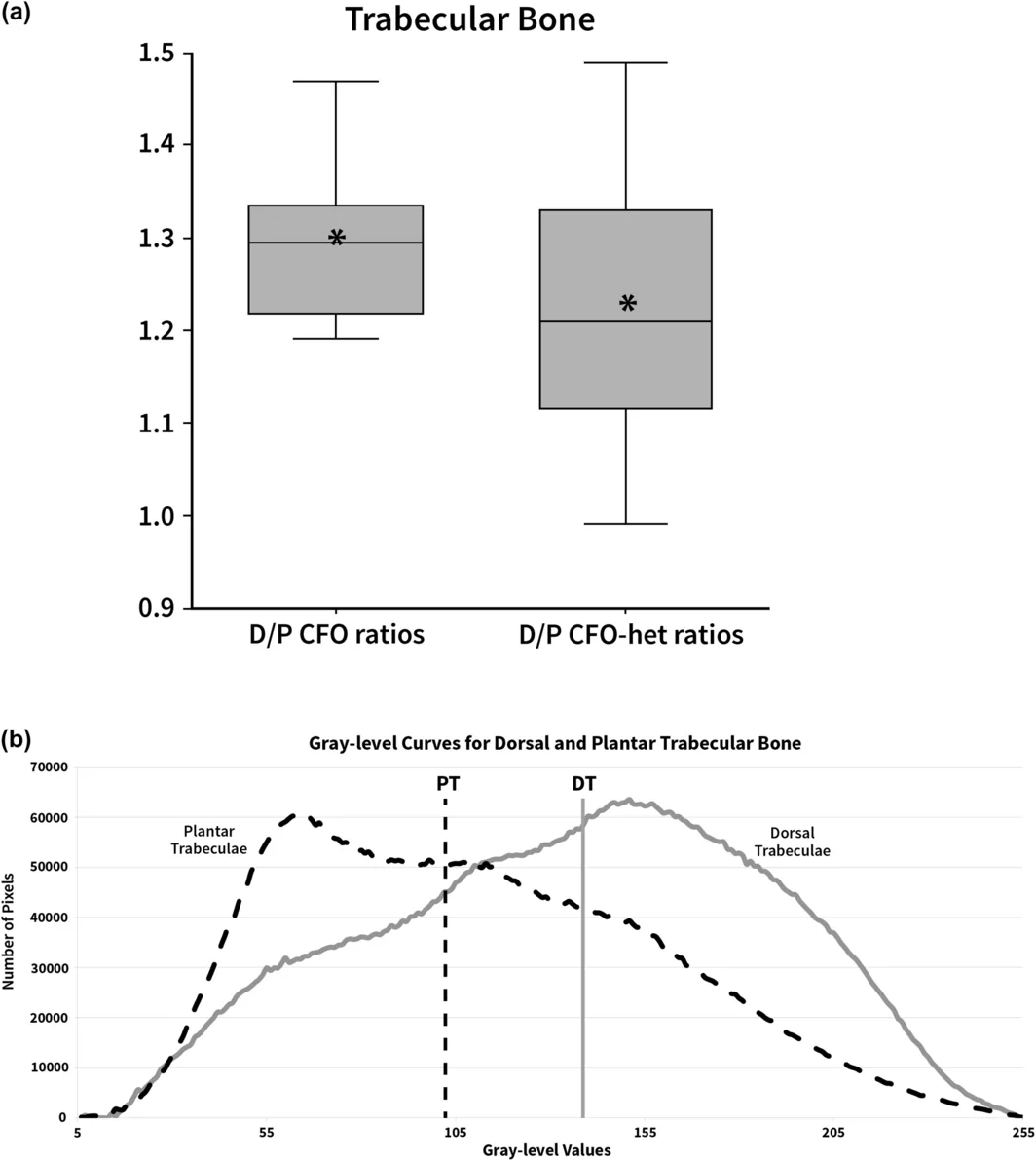
(a) Box and whisker plots of the dorsal/plantar (D/P) CFO/WMGL ratios and D/P CFO‐het ratios of the trabecular bone obtained from each animal. The lower and upper edges of the boxes represent the 25th and 75th percentile of the data, the medians are represented by asterisks, and mean values are the horizontal lines within the boxes. The whiskers represent the minimum and maximum ratios. (b) Gray‐level curves for the CFO/WMGL variations of the dorsal and plantar trabecular bone of an adult mule deer calcaneus. Notably, the dorsal trabeculae are significantly brighter (more oblique‐to‐transverse CFO, higher numerical values) than the plantar trabeculae and have a broader distribution of gray levels (greater CFO‐het) (*p* < 0.05). These curves are representative of the differences between the dorsal vs. plantar trabecular regions of all the bones used in the present study; all curves were unimodal. The vertical lines represent the WMGL values for the plantar trabecular bone (dashed black line, PT) and dorsal trabecular bone (solid gray line, DT).

The CFO/WMGL of each trabecular region tended to be more oblique‐to‐transverse than its adjacent cortex (*p* = 0.07–0.09). Hence, there is a tendency of more oblique‐to‐transverse CFO in each trabecular region when compared to its adjacent cortex: (1) 6% greater CFO/WMGL in the dorsal trabecular region than in the dorsal cortex, and (2) 7% greater CFO/WMGL in the plantar trabecular region than in the plantar cortex. Using the same method for the prospective sample size analysis, retrospective sample size analysis was done using the means and standard deviations of the CFO/WMGL data (see above) from the adjacent cortical and trabecular regions. This revealed that two additional specimens would be needed to detect a statistically significant regional difference in these comparisons.

As noted, qualitative observations in the confocal microscope showing few hemi‐osteon overlapped with small portions of adjacent hemi‐osteons support the conclusion that the regional differences in CFO‐het and predominant CFO between the trabecular tracts reported in this study occur at the individual hemi‐osteonal level of tissue organization.

In the correlation analyses conducted on all data from the dorsal and trabecular bone regions, there were two significant correlations: (1) CFO/WMGL and average mineralization showed a high positive correlation (*r* = 0.72, *p* < 0.01), and (2) CFO/WMGL and hemi‐osteonal packet prevalence showed a moderate negative correlation (*r* = −0.62, *p* = 0.02). In contrast, correlation analyses that considered all dorsal and plantar trabecular bone data revealed: (1) a low positive correlation between CFO/WMGL and CFO‐het, but this was not statistically significant (*r* = 0.48, *p* = 0.08), and (2) no statistically significant correlations between CFO‐het and the three non‐CFO material characteristics (mean mineral content, mineral content heterogeneity, and hemi‐osteonal packet prevalence) (all *p* > 0.3). When considering comparisons with only dorsal trabecular bone data, or with only plantar trabecular bone data, no significant correlations were found between CFO/WMGL, CFO‐het, and the non‐CFO characteristics.

## 4 DISCUSSION

Results of this study suggest that similar to secondary osteons in cortical bone, hemi‐osteons in trabecular bone may be able to adapt their collagen/lamellar organization in different regions for variations in strain mode/magnitude during peak loading. This is the first peer‐reviewed dorsal/plantar CPL analysis examining the possibility of strain‐mode‐specific adaptation of this material characteristic in trabecular bone in the deer calcaneus model. Prior studies in different bones and contexts (e.g., loading conditions and investigative methods) should be considered when interpreting our data from the deer calcaneus model. In particular, Osaki et al. (2009) mapped collagen orientation in the human calcaneus using a microwave method and demonstrated potentially mechanically meaningful patterns in the horizontal plane, but did not test for dorsal versus plantar strain‐mode relationships at mid‐shaft. Likewise, Ishimoto et al. (2018) showed preferential collagen–apatite co‐orientation and time‐dependent changes in trabecular bone in a vertebral implant model. Additionally, a study using synchrotron SAXS/WAXS tensor‐tomography (Grünewald et al., 2020) demonstrates that collagen‐associated nanostructure and apatite c‐axis orientation (i.e., preferential orientation of mineral crystallites) are often co‐aligned but can diverge locally (especially near cement lines/Haversian canals).

Although the regional CFO differences reported in the present study suggest that this is the dominant matrix‐level adaptation, there is the unexplored possibility that preferred mineral crystallite orientation can be mechanically advantageous in some load environments. This possibility should be explored using advanced technologies in three dimensions (3D) in both trabecular and cortical bone, especially when considering data reported by our group showing that potentially mechanically meaningful dissociations of predominant CFO and the orientation of extrafibrillar mineral crystallites can occur between the dorsal and plantar cortices of adult deer calcanei at mid shaft (Skedros et al., 2006). In that prior two‐plane analysis (only transverse and sagittal sections) of cortical bone, we posed the hypothesis that mineral crystallite orientation in the dorsal “compression cortex” might play a more significant role in accommodating compression, whereas in the plantar cortex, predominant CFO may have greater relevance in accommodating tension. Rigorous 3D analysis examining this intriguing possibility is needed in both trabecular and cortical bone.

The potential hemi‐osteonal collagen/lamellar adaptations reported here can be considered homologous to the differences in secondary osteon collagen/lamellar morphotypes that occur between the habitually compression‐ and tension‐loaded cortices of adult deer and sheep calcanei (Skedros et al., 2009). Similar findings suggesting strain‐mode‐specific CFO adaptation between opposing cortices have also been reported in other “types” of limb bones that are similarly differentially loaded in a predominant strain mode as the result of typically unidirectional bending (e.g., equine and sheep radii at mid diaphysis; human and chimpanzee femora at proximal diaphysis) (Riggs et al., 1993; Skedros et al., 2009; Skedros et al., 2013). As discussed below, we suggest that the CFO data from the present study and the other material‐level differences reported previously between the dorsal vs. plantar trabecular tracts of mature deer calcanei (Figure 2) could help accommodate differences in their strain environments—implying that it is possible that strain‐mode‐related adaptation can occur within the matrix of individual trabeculae. If this interpretation is correct, then has this observation also been made in other bones?

### 4.1 Lack of strain‐mode‐related CFO in putative tension versus compression trabecular tracts of human calcanei suggests more complex loading when compared to artiodactyl calcanei

Lozupone (1985) used polarized light to examine CFO patterns in microscopic images of thin transverse sections of individual trabeculae from each of the two main arched trabecular tracts in adult human calcanei. Many investigators consider one of these trabecular tracts to be predominantly loaded in tension and the other predominantly loaded in compression due to the simple unidirectional bending that is presumed to occur during typical walking/running ambulation (Bajraliu et al., 2016; Gefen & Seliktar, 2004; Giddings et al., 2000; Gierse, 1976; Saers et al., 2020; Saers et al., 2022a). However, Lozupone did not find evidence of the expected strain‐mode‐related CFO differences between these regions. The results of Lozupone's study contrast with the seemingly clear strain‐mode‐specific CFO differences (i.e., tension‐ vs. compression‐adapted) that we report herein between the dorsal and plantar trabecular tracts of deer calcanei. A possible explanation for the lack of regional tension‐ versus compression‐related differences between the main arched trabecular bone tracts of human calcanei is that it might simply reflect more complex loading patterns. There is relatively greater complexity/variability of loading of the hind foot in humans (Brockett & Chapman, 2016; Watari et al., 2021) compared to the simpler loading of artiodactyl ankle region, where conformation of the calcaneal/talocrural articulation is highly constrained (Skedros et al., 2019). Although the multidirectional/shear‐rich loading of the human calcaneus appears to be the primary explanation for the absence of simple strain‐mode‐specific patterns, there are also differing factors that may be involved (e.g., gait kinematics, soft‐tissue force transmission and dampening, and age/activity heterogeneity).

Although the artiodactyl calcaneus is described here as being more simply loaded than the human calcaneus, it is important to note that this relative simplicity of the artiodactyl calcaneus primarily applies to the central shaft region. In prior studies of mule deer calcanei, we described the central shaft region as the “pure bending region” and the distal shaft as the “mixed beam region,” indicating increased load complexity in the distal region (Sinclair et al., 2013; Skedros et al., 1994). Compared to the central calcaneal shaft, the distal calcaneal shaft contains a higher proportion of trabecular bone (the cortex becomes notably thin) and the cross‐sectional geometry is less elongated in the dorsal‐plantar direction. It is suggested that the quasi‐circular cross‐sectional morphology of the distal region accommodates increased load complexity (Skedros et al., 2023). For these reasons, the present study focused exclusively on the central portion of the calcaneal shaft, where loading is relatively simpler: dorsal‐plantar bending.

### 4.2 The dependence of CFO adaptation on strain mode and not strain magnitude

When a beam‐like structure like the artiodactyl calcaneus is subjected to bending, the outer cortical bone experiences the greatest tension and compression stress, while the trabecular bone inside and near the neutral axis experiences much lower stress. One might therefore expect that the large difference between the cortical and trabecular bone stress magnitudes would correspond to marked CFO differences. It might seem surprising, then, that the reported dorsal‐plantar differences between the cortical and trabecular bone regions are similar (31% cortical bone vs. 29% trabecular bone). However, these results are consistent with the idea that CFO‐related adaptations are dependent on strain mode rather than strain magnitude (Skedros, 2025a, 2025b; Takano et al., 1999). This is shown by the findings of the in vivo experimental study of Takano et al. (1999) in which they studied regional variations in CFO and peak strains in the radii from dogs in operated and sham‐operated groups (there are no other prospective controlled studies). They found that an experimentally induced change in the strain‐mode distribution (i.e., a “strain‐mode reversal”) in the operated group caused strain‐mode‐specific matrix formation; namely, more oblique‐to‐transverse CFO in the new “compression region” and more longitudinal CFO in the new “tension region.” Notably, the experimentally induced strain‐*mode*‐specific matrix changes did *not* correspond to the trans‐cortical variations in strain magnitude, but rather appeared to be responses to the strain *mode* that prevails across the entire cortex. Consequently, and despite lower mean stresses in the trabecular bone (due to its deeper location than the cortex), the matrix of the trabeculae shows CFO patterns that are consistent with strain‐mode‐specific adaptation, supporting the conclusion that strain mode is the dominant cue in this setting.

### 4.3 Comparisons with the human femoral neck emphasize the importance of considering influences of shear (in addition to compression and tension) in cases where loading is relatively complex

In a recent study, von Kroge et al. (2022) examined regional variations in predominant CFO in a small sample (*n* = 8) of thin sections of normal human femoral necks. They used methods similar to those described herein to analyze the trabecular tracts and adjacent cortices along the superior–inferior axis (presumably habitual tension‐ and compression‐loaded regions, respectively). Notably, they reported no significant differences in predominant CFO between the superior and inferior trabecular tracts, or between the superior and inferior cortices. They speculated that prevalent/predominant shear from multi‐directional loading of the human femoral neck might explain the lack of regional differences in CFO between the superior and inferior regions. In the human femoral neck, non‐orthogonal trabecular intersections (~70°) in the middle (“neutral axis”) region, as seen in both depictions in Figure 6, are believed to accommodate the deleterious effects of shear that can occur with predominant torsion/multidirectional loading (Boyle & Kim, 2011; Pidaparti & Turner, 1997; Reznikov et al., 2016). As described elsewhere (Richards et al., 2024; Skedros et al., 2023; Walker et al., 2023), this finding rejects the conventional idea that the human femoral neck region often experiences simple cantilever‐type loading. Conversely, similar non‐orthogonal trabecular intersections are not seen in 2D analyses of deer and sheep calcanei (Skedros & Baucom, 2007) (Figure 1). If shear‐related adaptation is at play in the material organization of trabecular bone of mule deer calcanei, then it might be most obvious where the tracts extensively intersect (i.e., at the neutral axis and distal shaft regions) because this is where shear stresses are the highest (Sinclair et al., 2013; Skedros et al., 2019). Unfortunately this possibility could not be explored in the present study because the neutral axis (middle) region was not sampled. More extensive examination of trabecular bone of the deer calcaneus (especially in the middle region) at the microscale and nanoscale in 3D, coupled with mechanical testing, is needed to advance its utility as a model for evaluating adaptations to regional variations in the prevalence of a particular strain mode.

**FIGURE 6:**
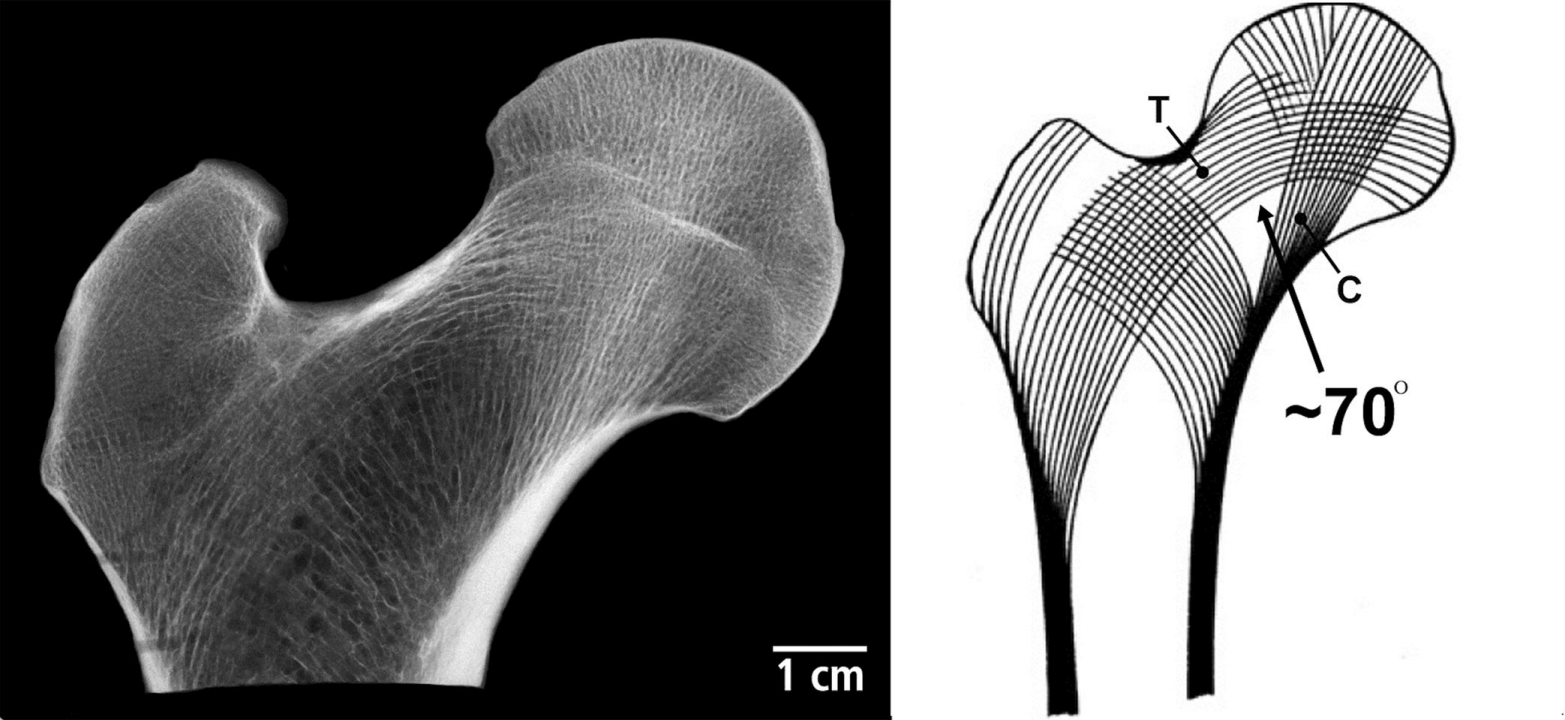
At left is a radiograph of a thin frontal section of a human proximal femur from a 42‐year‐old male. Because typical ambulation results in a downward force on the hip, it is typically assumed that net tension (T) occurs across the upper (superior) neck region and net compression (C) along the inferior neck region (as shown in the drawing at right). This conventional understanding of the habitual loading of the hip is believed to be reflected in the arched trabecular patterns, as if they resemble tension and compression stress trajectories (Skedros & Baucom, 2007; Walker et al., 2023). At right is von Meyer's 1867 drawing showing two‐dimensional depictions of arched trabecular tracts with added labels (T = tension tract, C = compression tract) (Skedros & Brand, 2011; von Meyer, 2011). As described in the text, the *non*‐orthogonal trabecular intersections (~70°) in both femora depictions are believed to accommodate the deleterious effects of shear that can occur with prevalent/predominant multidirectional loading. In contrast, orthogonal trabecular intersections are present in deer and sheep calcanei (Figure 1) (Reproduced from Skedros et al. (2023) with permission of John G. Skedros).

### 4.4 Some micromechanical functions of typical osteon and hemi‐osteon collagen/lamellar variations (“morphotypes”) are likely similar

The mechanical benefits of conventional osteons (i.e., secondary osteons) in cortical bone have been studied extensively (reviewed in Currey, 1984; Skedros, 2025b). Results of many of these prior studies can be extrapolated to help understand the mechanical benefits of hemi‐osteons in trabecular bone, which is helpful because of the paucity of studies in this context (we are not aware of such studies with respect to hemi‐osteonal collagen/lamellar morphotypes). Based on data from healthy adult humans and nonhumans, it is speculated that osteon collagen/lamellar morphotypes in cortical bone (these are not hemi‐osteons) reduce stress fracture risk by differentially toughening regions of the same bone for tension strains in some locations and compression strains in others (Nunamaker, 2001; Skedros et al., 2013). This regional microstructural toughening in cortical bone is likely strongly influenced by osteonal remodeling that produces regional variations in population densities of osteon collagen/lamellar morphotypes and their associated interfaces (e.g., cement lines and interlamellar seams). These interfaces, microscopic mineralization variations, CFO variations, and/or lamellar patterns/heterogeneity introduced by osteons enhance the tissue's capacity to attenuate microcrack propagation, which helps to extend fatigue life, thus avoiding fracture (Ciarelli et al., 2009; Gibson et al., 2006; Katsamenis et al., 2015; Martin & Burr, 1989; Reilly et al., 1997; Reilly & Currey, 1999; Yeni & Norman, 2000). The significant regional variations and correlations shown in the present study between predominant CFO and tissue mineralization and hemi‐osteonal packet prevalence might similarly reflect fracture‐resistance mechanisms at the nanostructural and microstructural levels. Such biomechanically relevant associations that have been shown in cortical bone would be expected in trabecular bone (Torres et al., 2016).

Independent of osteons, the integrity of the collagen network (e.g., variations in collagen cross‐links, predominant CFO, CFO‐het, lamellar variations, etc.) in bone tissue is considered integral for adequate bone toughness, and degradative changes in many such non‐osteonal characteristics have been implicated in age‐related decreases in toughness in human cortical and trabecular bone (Burr, 2011; Torres et al., 2016; Wang et al., 2002; Willett et al., 2022; Zimmermann et al., 2015). We found that CFO‐het was significantly greater in the dorsal trabecular region compared to the plantar trabecular region. It would be expected that more oblique‐to‐transverse CFO in predominantly compression‐loaded regions would be correlated with greater CFO‐het (because CFO and CFO‐het are likely related in this context [*r* = 0.48, *p* = 0.08]). The idea that CFO‐het helps to enhance tissue toughness is supported by the fact that CFO‐het is a prominent characteristic of deer antlers, which have extraordinary toughness (Currey, 1984; Dakoulas et al., 2011; Skedros et al., 2014; Skedros, Keenan, et al., 2011). Hence, it is conceivable that the regional differences in CFO heterogeneity (i.e., greater dorsally) reported in the present study may enhance toughness by deflecting cracks across orientation gradients (Katsamenis et al., 2015).

### 4.5 Collagen fibers vs. fibrils and other characteristics of trabecular bone at the microscale and nanoscale in the perspective of extrinsic and intrinsic toughening mechanisms

To understand the mechanical relevance of the regional variations in hemi‐osteonal morphotypes, it is also important to distinguish the potential influences of collagen fibrils in addition to collagen fibers. Collagen fibrils are composed of tropocollagen molecules and hydroxyapatite nanocrystals combined into thread‐like structures. Collagen fibrils are then woven together into larger helical structures that form collagen fibers (Alexander et al., 2012; Holmes et al., 2018; Launey et al., 2010). Consequently, adequate evaluation of collagen *fibrillar* alignment requires use of much thinner sections than those used in this study (e.g., <5 μm) and methods that provide much higher resolution than the conventional polarized light used in the present study. Advanced methods that would allow for nanoscale resolution and would provide more quantitative, accurate, and novel measures of collagen fibrillar alignment/organization (and also mineral characteristics) in bone than polarized light microscopy include focused ion beam scanning electron microscopy (FIB/SEM), transmission electron microscopy (TEM), submicron computed tomography (μCT), x‐ray microscopy, polarized Raman confocal microscopy/spectroscopy, and others (Alexander et al., 2012; Buss et al., 2024; Georgiadis et al., 2016; Raguin et al., 2021; Schrof et al., 2014; Starborg et al., 2013).

However, there are advantages in obtaining averaged CFO data using CPL in relatively large specimens even though it is a semi‐quantitative method (Boyde & Riggs, 1990; Bromage et al., 2003; Skedros, 2025b). Regional variations in CFO are readily apparent at the selected section thickness of 50 μm and this: (1) is within the range (30–60 μm) recommended by Spiesz et al. (2011) in a rigorous study aimed at achieving more quantitative, and calibrated, CFO data when using CPL microscopy, and (2) approximates the standard thickness of 100 μm used for analyzing CFO in relatively large regions of a bone cross‐section using polarized light microscopy (Boyde & Riggs, 1990; Bromage et al., 2003; Nakai & Boyde, 2024). Because the focus of the present study was on predominant CFO patterns in relatively large regions of a bone, the thickness of the sections was also appropriate when considering that it resembles the type of analysis that has been done on bulk specimens used in mechanical testing of different regions of deer calcanei and equine third metacarpals that are loaded naturally in a predominant strain mode (Skedros, Dayton, Bloebaum, et al., 2024; Skedros, Dayton, Cronin, et al., 2024).

### 4.6 Limitations of this study, and the potential coupling of material/tissue‐level and structural/architectural characteristics in trabecular bone

One limitation of the present study is that trabecular CFO patterns were examined only in the transverse plane (with respect to the long axis of the bone). Trabeculae would be expected to be distributed in 3D in an anisotropic manner; hence, tension or compression stresses imparted to bulk regions of trabecular bone would have a complex stress distribution when compared to nearby cortical regions. Consequently, the differences in net tension and compression between the respective plantar and dorsal regions would not be as distinct as we assume. However, in our prior studies where we quantified eight trabecular architectural characteristics in the dorsal and plantar trabecular regions of adult mule deer calcanei, only one of these (trabecular number) showed a probable mechanically relevant dorsal/plantar difference (but this dorsal/plantar difference was not found in trabecular bone of adult sheep calcanei) (Sinclair et al., 2013; Skedros et al., 2012). Thus, when considering the various typically studied architectural characteristics of trabecular bone in these artiodactyl calcanei, there is no clear relationship with the dorsal and plantar trabecular regions despite their differences in load history. While this suggests that the CFO patterns reported in the present study are not confounded by variations in trabecular architecture, it does not rule out that possibility.

Consequently, studies of CFO are needed in the dorsal and plantar regions in various section planes and with consideration of potential relationships with variations in trabecular architectural characteristics (not all architectural characteristics have been studied in the artiodactyl calcaneus model). Prior studies that have considered the possibility of structural‐material coupling in trabecular bone are few and have been conducted in regions where material/tissue‐level characterizations have been limited (e.g., only mineralization variations have been studied), load‐history is unclear, and/or age effects are present and exceedingly difficult to control (e.g., these studies are typically in human bones) (Lamarche et al., 2025; Torres et al., 2016; Yu et al., 2021). Additionally, the work of Bidan and colleagues (Bidan, Kommareddy, et al., 2013; Bidan, Wang, & Dunlop, 2013) and Deering et al. (2025) suggests that the geometry of the precursor substrate may influence the orientation of collagen fibers when new extra‐cellular matrix is laid down. This potential effect on overall CFO patterns should be considered in future work on trabecular CFO patterns in the artiodactyl calcaneus.

Another important limitation of this study is that the “tension trabecular tract” and “compression trabecular tract” of the artiodactyl calcaneus, or any other bone with similar arched trabecular patterns, cannot function independently because they are highly interconnected (Figures 1 and 6). Although we sampled bone in regions where the tracts appear to become independent when viewed in lateral radiographs, this confounding issue cannot be eliminated. It is also possible that strains measured on the surface of a bone might not closely reflect the magnitudes and/or distributions of the strains produced in the deeper trabecular bone (Bay et al., 1999; Keaveny et al., 2001; Renders et al., 2006; Van Rietbergen et al., 2003). We know of no evidence supporting or rejecting the assumption that the dorsal and plantar tracts experience a different and habitually predominant strain mode.

A final limitation for consideration is that our findings are based on only one species, which raises the possibility of a species‐related difference that might not be generally applicable to similar bones in other cursorial species (or in different anatomical sites). Additional studies in diverse models are necessary to confirm whether strain‐mode‐specific CFO adaptation in trabecular bone is a general phenomenon that occurs in bones and bone regions where habitual relatively unidirectional bending is present. These additional studies are needed to more rigorously determine the basis for our positive findings and the negative findings in the aforementioned human studies.

## 5 CONCLUSIONS

Results of this study demonstrate what appears to be evidence of the capacity of trabecular bone to exhibit material‐level adaptation that enhances mechanical properties for specific load environments in ways that structural characteristics alone cannot achieve. Mechanical testing studies are needed to test this hypothesis. Future studies are also needed at the micro and nano scales to evaluate the composition and biomechanics of the trabecular bone of the artiodactyl calcaneus model, especially for regional variations in 3D collagen fibrillar alignment/organization.

## AUTHOR CONTRIBUTIONS

**John G. Skedros:** Conceptualization; investigation; funding acquisition; writing – original draft; methodology; validation; visualization; writing – review and editing; data curation; supervision; resources; formal analysis; project administration; software. **John T. Cronin:** Conceptualization; investigation; writing – original draft; writing – review and editing; formal analysis. **Chad S. Mears:** Conceptualization; investigation; writing – original draft; methodology; writing – review and editing; validation; formal analysis. **Brett W. Richards:** Conceptualization; writing – original draft; writing – review and editing; validation; investigation.

## CONFLICT OF INTEREST STATEMENT

The authors have nothing to disclose.

## Data Availability

All data can be accessed via correspondence with the corresponding author.
